# Behavioral Management as a Coping Strategy for Managing Stressors in Primates: The Influence of Temperament and Species

**DOI:** 10.3390/biology11030423

**Published:** 2022-03-10

**Authors:** Sierra Palmer, Scott Hunter Oppler, Melanie L. Graham

**Affiliations:** 1 Department of Surgery, University of Minnesota, Minneapolis, MN 55108, USA; palme535@umn.edu (S.P.); opple001@umn.edu (S.H.O.); 2 Department of Veterinary Population Medicine, University of Minnesota, St. Paul, MN 55108, USA

**Keywords:** training, nonhuman primates, coping, temperament, behavior, welfare

## Abstract

**Simple Summary:**

Species, motivation, and temperament are characteristics that influence environmental perception and learning in animals, and consequently, welfare. We investigated the relationship between these individual traits and training success in primates to acquire skills for cooperation and participation with medical care including sample collection, drug administration, vitals monitoring, and examination. Despite behavioral differences related to temperament, all animals successfully completed the training program without significant differences in time required to acquire target skills. Training time was significantly different between rhesus and cynomolgus macaques, likely reflecting species differences in memory, motivation, reasoning, and learning. However, with the perspective of typical study duration and long lifespan in primates, this difference in time to completion was clinically irrelevant. A well-designed training program that is properly applied can establish positive coping skills in primates across temperament and other traits to strengthen psychological resilience, improving welfare and reducing stress confounding for more accurate scientific translation.

**Abstract:**

Primates involved in biomedical research experience stressors related to captivity, close contact with caregivers, and may be exposed to various medical procedures while modeling clinical disease or interventions under study. Behavioral management is used to promote behavioral flexibility in less complex captive environments and train coping skills to reduce stress. How animals perceive their environment and interactions is the basis of subjective experience and has a major impact on welfare. Certain traits, such as temperament and species, can affect behavioral plasticity and learning. This study investigated the relationship between these traits and acquisition of coping skills in 83 macaques trained for cooperation with potentially aversive medical procedures using a mixed-reinforcement training paradigm. All primates successfully completed training with no significant differences between inhibited and exploratory animals, suggesting that while temperament profoundly influences behavior, training serves as an important equalizer. Species-specific differences in learning and motivation manifested in statistically significant faster skill acquisition in rhesus compared with cynomolgus macaques, but this difference was not clinically relevant. Despite unique traits, primates were equally successful in learning complex tasks and displayed effective coping. When animals engage in coping behaviors, their distress decreases, improving welfare and reducing inter- and intra- subject variability to enhance scientific validity.

## 1. Introduction

The ability to effectively cope with stressful situations has a major impact on physical and psychological wellbeing in both animals and humans. Coping consists of strategies and behaviors used to manage situations that are perceived as stressful, divided into two types, positive (e.g., creation of a favorable association or reappraisal) or negative (e.g., avoidance and escape) [1]. Chronic, uncontrolled stress in the absence of effective coping has been associated with an increased risk of developing anxiety, depression, and a range of other disorders [2,3,4]. In the healthcare setting, anxiety or depression, as well as overall patient dissatisfaction, can increase the likelihood of poor compliance with drug or treatment regimens, negatively impacting a variety of patient health outcomes [5,6]. In contrast, positive coping has the potential to reduce distress associated with illness and aversive medical interventions to the extent that effective coping strategies have been shown to improve patient quality of life (QOL) as well as decrease morbidity and mortality [7,8,9,10,11]. A combination of appropriate cognitive and behavioral responses is necessary in order to reinterpret, or blunt, aversive events or demands imposed by stressors and effectively cope [12,13]. Animals involved in biomedical research are intended to closely model diseases and therapies under investigation, which not only exposes them to similar stressors that affect patient quality of life (i.e., inherently imposed by a specific disease state and its intensive medical management) but also those related to the introduction of frequent research interventions in a captive environment. Even with the most skilled care and appropriate pain management, if animals are not adequately prepared these interventions can be perceived as unpredictable and uncontrollable. As a result, interactions with an aversive stimulus can have a variety of behavioral consequences including conditioned anxiety, attempts to avoid or escape treatment, or direct aggression towards caregivers. The frequency of medical intervention can intensify distress as animals do not necessarily habituate to aversive procedures simply through repetition alone. Ultimately, the ability to cope with these stressors impacts animal welfare and scientific outcome parameters [14,15,16,17,18].

In the captive setting, behavioral management is a comprehensive approach combining enrichment, sociality, and training to enhance welfare and foster positive coping [19]. Operant conditioning is an important and frequently used tool in learning and behavioral modification for both routine situations and stressful situations where avoidance learning can lead to maladaptive coping. In the research setting, a number of benefits have been observed in animal models trained to cooperate with their own care, including safer animal-caregiver interactions, improved model validity from reduced levels of outcome-confounding stress, and enhanced welfare [20,21,22,23,24]. Likewise, in pediatric patients, the use of behavioral management techniques that incorporate the opportunity to express choice successfully fosters beneficial cooperative behavior during aversive treatments, highlighting the translational relevance of these paradigms [25,26].

The success of any training paradigm is dependent on the acquisition of specific skills and the speed, or efficiency, of learning. Positive reinforcement training (PRT), defined as ‘adding’ a rewarding stimulus (e.g., preference food item, toys, positive social interaction) upon performance of a desired behavior, is generally considered the ideal training approach [21]. However, PRT alone has been shown to be less effective for training behaviors that have mildly aversive outcomes (e.g., pain from a blood collection or drug injection) and require animals to make a value assessment of whether a reward is ‘worth it’ [27,28]. Though the connection is not well understood, prior research has suggested that temperament is correlated with successful training of simple tasks and can influence an individual’s relative predilection for appetitive versus defensive (e.g., avoidance) motivators, affecting eventual cost–benefit decisions and the overall efficacy of PRT-alone training paradigms [29,30]. Primates with more inhibited temperaments, with behavioral tendencies towards withdrawal or apprehension of the unfamiliar [31], have been shown to have a more difficult time learning simple tasks under a PRT-only paradigm compared to primates with exploratory temperaments, suggesting that inhibited animals may place a greater value on avoidance over reward [32]. When PRT alone is ineffective in suppressing avoidance motivated behavior, a concurrent negative reinforcement training (NRT) component, usually a mild unwanted condition (e.g., reduced working space, elimination of additional sessions when animals accomplish behaviors to promote productivity), can be used intentionally and selectively (as opposed to accidentally) because of the inherent aversive stimuli occurring in most medical situations [33]. Giving animals the opportunity to rehearse the behavior necessary for cooperation or coping with procedures in stepwise in a highly controlled safe environment alters experience from an undesirable one to a tolerable or even a sought after experience when effectively converted to reward seeking. This strategy, desensitization, increases familiarity with a task, while reducing fear, to help decrease overall stress related to the task and can foster the expression of choice and cooperative behavior, indicators of successful coping, in future instances of the task [21,34,35].

Coping has also been linked with temperament, or personality [36,37]. Temperament encompasses the consistent emotional and behavioral traits of an individual that are a major factor influencing the subjective environmental experience [38,39,40,41,42]. The dimensions of temperament and personality are remarkably similar across species [43,44]. Rodent temperament has been used to predict anxiety traits and evaluate physiological mechanisms related to psychopathology [45,46,47,48], while nonhuman primate (NHP) behavior, social constructs, cognitive function, and temperament-defining traits are each closely related to those of humans [49,50,51,52,53]. Temperament influences reactivity to acute and chronic stress, manifesting as changes in physiological parameters such as heart rate, blood pressure, and endocrine response [54,55,56], and has been associated with overall response to stressful clinical situation [38,57]. Since temperament and personality influence how individuals perceive their environment and cope with its stressors, there is likely a similar influence on individual responses to targeted interventions aimed at improving coping and fostering resilience [29,36,37,58,59,60], including various behavioral training paradigms [36]. Temperament has been shown to indirectly influence learning by affecting motivation and preference in learning styles [61,62,63,64]. As such, mixed reinforcement paradigms may have the considerable advantage of motivation across temperament to support wider-spread acquisition of positive coping across all temperament types [65,66,67].

While training animals for cooperation in biomedical research is becoming more common, few studies have examined factors that might influence the success of such behavioral management programs. Using our well-established training program designed to foster NHP cooperation with a variety of research and medical interventions, we evaluated the influence of individual characteristics such as temperament and species on training outcomes and the acquisition of important coping skills [20]. This retrospective cohort study assessed training success in male and female rhesus (*Macaca mulatta)* and cynomolgus macaques (*Macaca fascicularis*) with inhibited and exploratory temperaments. Animals were trained using a standardized mixed reinforcement paradigm [68] to cooperatively present a limb to a caregiver in their familiar home enclosure for the performance of a variety of medically relevant manipulations. While holding environmental and social conditions constant, we probed the influence of temperament, species, sex, and age on the time required for animals to acquire the targeted skills. We defined success as full cooperation with caregivers with the target task, suggestive of a motivational preference towards appetitive, rewarding stimuli relative to avoidance. In human patients, behavioral engagement, active and voluntary participation, and the need for restraint (or lack thereof) have similarly been used to assess coping in the context of medical-related stimuli in human patients [66,69,70]. Similarly, we considered engagement and voluntary participation with direct human contact and medical stimuli indicative of positive coping. There is important translational relevance in this work, especially in pediatrics, where reactivity in children is particularly closely tied to temperament due to developmentally primitive coping mechanisms [71,72]. This study evaluated the relationship between temperament, species, and other demographic variables and the acquisition of coping skills with the aim to both improving captive animal management practices and also reveal novel insights into the interplay between temperament and coping that can inform strategies in the clinic [73,74].

## 2. Materials and Methods

### 2.1. Animal Subjects

All animal use was approved by the University of Minnesota Institutional Animal Care and Use Committee, was in compliance with the Animal Welfare Act, and adhered to the principles stated in the Guide for Care and Use of Laboratory Animals. 

A total of 51 healthy rhesus (female = 24, male = 27) and 32 healthy cynomolgus (female = 11, male = 21) macaques, ages ranging from 1.5 to 13 years (median = 4.0 years), were enrolled in this study (Table S1). All animals were purpose-bred and acquired from institutionally approved commercial vendors. Animals were concurrently enrolled in separate metabolic studies as part of a preclinical research program. All animals were housed in same-sex pairs or groups, except in rare cases of demonstrated social incompatibility. Water was available ad libitum, and primate biscuits (2055c or 7195 Envigo Harlan Teklad Nonhuman Primate Diet or 5048 LabDiet Certified Primate Diet) were provided twice daily based on body weight and supplemented with additional food enrichment consisting of fresh fruits, vegetables, grains, beans, and nuts. All animals participated in an environmental enrichment program that included novel toys, music, social play and regularly scheduled access to a large play and exercise area with swimming access. Room temperature was maintained at 20–26.7 °C, humidity was maintained at 30–70%, and lights were programmed to a 12 h-on (5:30 a.m.–5:30 p.m.), 12 h-off circadian light cycle with 30 min dawn/dusk intervals. Animals were observed at least twice daily for general appearance, behavior, and body condition as a part of routine health monitoring. Semi-annual veterinary physical examinations were performed and included assessments of weight, body condition, heart rate, temperature, lymph nodes, abdomen, oral cavity, dermis, ears, and nose, as well as the performance of a complete blood count and chemistry panel. Weights were taken at least monthly, and veterinary rounds for routine evaluation were performed weekly. 

### 2.2. Primate Temperament Assessment

Primate behavioral traits and temperament were assessed from each animal’s home enclosure at the time of admission to the facility. Each animal’s behavior was documented by experienced trainers over a duration of approximately 10 min. Additionally, each primate was offered high-value food items by hand from these unfamiliar trainers in order to assess their willingness to take high-value appetitive items from a stranger. Study directors used the observed behaviors to evaluate and classify each animal’s temperament as either “inhibited” or “exploratory”. To do this, observed behaviors were classified as either inhibited-type, exploratory-type, or neutral-type; behaviors that are accepted as highly characteristic of either exploratory or inhibited animals were given greater weight during this evaluation process. A sample list of typical behaviors, and their respective temperament associations, is provided in Table 1. Generally, animals that exhibited behavioral tendencies of being bold, curious, and willing to explore novel items were classified as exploratory, while animals with behavioral tendencies indicative of being fearful, avoidant, and easily disturbed by the unfamiliar were classified as inhibited [75]. The temperament classifications of each animal were validated through subsequent blind scoring by the principal investigator of the laboratory.

### 2.3. Primate Training

Primates were trained to accept oral fluids and foods, shift on cue, and voluntarily present a limb to facilitate the performance of medically relevant manipulation. The specific target behavior was defined as cued lower limb presentation by the animal via an opening in the home enclosure and remaining stationary in hold for cooperation with a range of commonly performed clinical manipulations such as sample collection (heel stick, intravenous catheter placement, vascular access port access), drug administration (subcutaneous (SC) or intramuscular (IM) injection), vitals monitoring, and examination. For each animal, training was performed by multiple trainers using a standardized, mixed reinforcement paradigm. The training program was divided into three distinct phases, referred to as P(re)-Phase, Phase-1, and Phase-2, each consisting of multiple sequential subphases; animals performed training sessions within a given subphase until successfully completing the subphase’s predefined objectives. Early subphases of training incorporated a combination of both positive reinforcement (PR) and negative reinforcement (NR), while later subphases transitioned to PR-only. Application of NR was strategically used to reduce and extinguish avoidance and escape-type behaviors associated with neophobia. In these early stages of training, a panel that animals had previously been desensitized to was used to limit enclosure space and bring the animals into closer proximity to the trainer; once animals were in close proximity, were relaxed and began to accept treats, the panel was removed (NR) and an additional jackpot (highly preferred item, e.g., cupcake or popsicle) reward was given (PR). In this context, the application of NR along with conventional PR facilitated all animals being able to receive and experience appetitive rewards in a highly controlled manner, as opposed to having the opportunity to continuously avoid both close proximity to the trainer and the reception of appetitive rewards [33].

The objective of the first phase of training, P-phase, was to desensitize the animal to both close contact with trainers and to the enclosure’s squeeze-back panel, and to associate trainers with rewards. Desensitizing the animal to the squeeze-back panel allowed for its future use as a neutral tool (related to space manipulation versus its conventional purpose for restraint) in subsequent training phases without risk of inducing fear, and to encourage acceptance of preferred treats by hand from trainers in order to strengthen the reward value of treat-giving as a form of PR in subsequent training phases. P-Phase consisted of 5 subphases, described below, each designed to gradually build on the skills acquired in the previous subphase to achieve these objectives. 

Each P-Phase training session occurred with the animal positioned in an area of the home enclosure planned for training and working. Each of the P-Phase subphases followed the same basic structure with slight variations, described below. At the start of each session, the animal’s affective state and attitude (e.g., engaged, neutral/calm, curious, anxious) were evaluated; if an animal was identified as being highly anxious or aggressive, the session would be stopped or not initiated for the evaluation of potential simple environmental factors that could be considered to better accommodate the individual animal’s engagement with the session. In these situations, some examples of possible adaptations include adjusting to the animal’s preference towards male or female trainer, time of day, or performance order of training in the room. Following attitude assessment, a variety of highly palatable treats, such as fresh or dried fruit, fruit snacks, assorted nuts, or assorted cereals were offered to the animal. Treats were initially offered by hand at the front of the enclosure, close to the location where the animal would be positioned for limb presentation in subsequent training phases. If the animal rejected taking any treats by hand, other offering methods were attempted: first, a treat was placed at the front of the enclosure with the trainer present; if the treat was still not accepted, it was finally offered at the front of the enclosure with the trainer out of the animal’s sight. Following initial treat offering, regardless of treat acceptance, the main part of the session began: treats were offered to the animal at least once every 20–30 s, with increased frequency if the animal accepted the treats and remained in the desired target location in its enclosure. A single session continued until either the animal had taken treats by hand 9 different times, or 4 min had elapsed since the start of the session, whichever occurred first. At the end of the session, the animal’s affective state and attitude were again evaluated, and a final treat was offered to the animal by hand. 

At the end of each P-Phase training session, a score was assigned to determine whether the animal adequately met the objectives of the session to be able to move on to the next subphase during the next training session. When an animal did not meet the required score to advance, the same subphase was repeated for the next training session. In the first subphase of P-Phase (P-1), up to 2 points could be assigned: one point was given if the animal took a treat from the trainer’s hand at the end of the session, and one point was given if the animal demonstrated a neutral or positive attitude (e.g., engaged, calm, or curious) at the end of the session; each of the two points were required to advance to the next subphase. Training sessions for subphases P-2, P-3, and P-4 followed the same attitude assessment and treat-offering procedures described above with a gradual increased incorporation of the enclosure’s squeeze-back panel. After the same start-of-session attitude assessment and treat offering, subphase P-2 was performed with the panel taking up 30% of the enclosure’s space, subphase P-3 was performed with the panel taking up 50% of the enclosure’s space, and subphase P-4 was performed with the panel taking up 90% of the enclosure’s space. Scoring and passing criteria for subphases P-2 through P-4 were similar to that used for P-1, but score was based on attitude and willingness to take offered treats by hand throughout the session, rather than the end of the session (as was carried out for P-1). 

The final subphase of P-Phase, P-5, was designed to introduce the animal to contact with the trainer. Each P-5 session began with a start-of-session attitude assessment and treat offering, followed by incorporation of the squeeze-back panel to take up 90% of the enclosure (the same position as in P-4). Once the squeeze-back was engaged, the trainer intermittently touched the animal’s toes within the enclosure or attempted to gently hold their leg for a maximum of 10 consecutive seconds per intermittent attempt. Treats were offered over the duration of the session. Each P-5 training session continued until either the animal had a non-reactive response to toe-touching and/or limb holding three different times, or 4 min had elapsed since the start of the session, whichever occurred first. At the end of the session, the animal’s affective state and attitude were evaluated. Similarly to other subphases, each P-5 session was scored based on attitude and treat taking during the session. With respect to attitude, an animal received a score of 2 points if it was engaged, neutral/calm, or curious during the session, 1 point if it was submissive or tentative, and 0 points where anxious or aggressive attitude/behavior was observed. An additional point was assigned if the animal took at least one treat by hand during the session. Two of the three possible points were required to complete P-Phase training and advance to Phase 1 of the training paradigm. Upon completion of the entire P-Phase, an animal had demonstrated the ability to both calmly take treats from the trainer with the squeeze-back panel engaged and allow the trainer to touch their toes and legs. A complete summary of P-Phase training steps, including a breakdown of session-end criteria and session passing-criteria is provided in Table 2. 

The objective of Phase-1 of the training paradigm was to familiarize each animal with voluntary limb presentation through an opening in their home enclosure for handling while remaining calm and stationary. Counterconditioning was used to associate typical stimuli animals encounter during intensive medical management with positive events (highly preferred food reward and control over the environment and space) and to associate these encounters as ‘safe’ with trainers. In Phase-1, the amount of space restricted by the squeeze-back panel was gradually decreased through subsequent subphases. In the latter subphases of Phase-1, each animal was also acclimated to handling techniques required for routine clinical evaluation, including ‘skill sets’, consisting of light toe hold, handler hand switch, foot squeeze, startle, knee flex/extend, leg movement on axis, skin pinch (for evaluating skin turgor/hydration), heel tap and squeeze (simulating heel stick), and needle sticks. Following the successful completion of Phase-1, animals advanced to Phase-2, with the objective of transitioning to voluntarily present their limb on cue with different trainers. At the end of each Phase-1 and Phase-2 training session, the animal was presented with a jackpot to mark the achievement of a complicated behavior. Throughout all Phase-1 and Phase-2 training sessions, total session time, hold time, and number of animal escape attempts or aggression were recorded. Training was designed to introduce new behaviors and skills at pace with the development of coping, evident in relaxed and cooperative task performance; animals that displayed ineffective coping, including signs of anxiety such as avoidance, escape attempt, alarm reaction, or aggressive behavior required repetition of a session in the current subphase. This ensured that animals were not exposed to high stress or overwhelmed and only given a progressively more difficult task as coping developed during a simpler and perceptively less-threatening task. Complete details of Phase-1 and Phase-2 training methodology as used in this study have previously been described by Graham et al. [68].

### 2.4. Data Analysis 

The relationship between primate temperament, species, age, and sex with various training outcome measures was assessed using independent samples t-tests, logistic regression, and Kaplan–Meier time-to-event analysis. Logistic regression was used to assess the predictive value of age, species, and sex in association with temperament on training success and willingness to take treats. For logistic regression analysis, age and training time were categorized as binary variables: for age, animals were classified as “young” or “mature” based on whether the animal was above or below the mean age of the entire cohort (4.3 years); for training time, animals were classified as “slower” or “faster” based on whether time to complete training was above or below the average total training time for the cohort. Kaplan–Meier time-to-event analysis was used to assess differences in time-to-training completion for the entire training paradigm and individual training phases, stratified by species and temperament. Overall differences in training times by species were further investigated using independent sample t-tests.

## 3. Results

Of the 83 primates assessed for temperament, 29% were classified as inhibited, and 71% were classified as exploratory (Table S1). Traits including sex, age, species, and temperament were analyzed to determine if there were differences in skill acquisition or coping. All primates (100%) in this study successfully completed all components of the training paradigm. At the conclusion of training animals consistently demonstrated the ability to voluntarily present a lower limb through an opening in their home enclosure, cooperate with handling, and remain relaxed during a potentially mildly aversive medical manipulation, consistent with positive coping.

We analyzed the willingness to take treats from unfamiliar trainers between exploratory and inhibited animals during their initial temperament assessments to investigate the relationship between temperament and novelty seeking. Cynomolgus macaques that were categorized as inhibited were less likely than exploratory animals to take treats from an unfamiliar trainer at time of admission to the facility (OR = 0.08, 95% CI = (0.01, 0.44), *p =* 0.01) (Table S2). Similar comparison was not performed for rhesus macaques due to insufficient data on treat acceptance to adequately power the analysis. 

**Figure 1:**
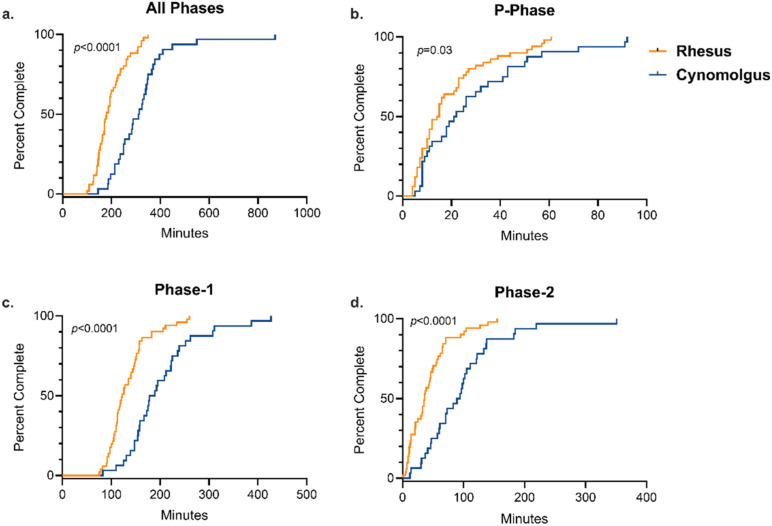
Training Completion Time by Primate Species. Kaplan–Meier time-to-event analysis comparing the time required to successfully complete all phases and individual phases of the training paradigm between rhesus and cynomolgus macaques. There was a significant difference between species for the completion of (**a**) all phases (Log-rank χ^2^ = 31.6 (df = 1), *p* = <0.0001), (**b**) P-Phase (Log-rank χ^2^ = 4.2 (df = 1), *p* = 0.04), (**c**) Phase-1 (Log-rank χ^2^ = 22.3 (df = 1), *p* = <0.0001), and (**d**) Phase-2 (Log-rank χ^2^ = 18.7 (df = 1), *p* = <0.0001).

We compared both individual phase and total training time across rhesus and cynomolgus macaques to assess the relationship between species and training success. Cynomolgus macaques took significantly longer, on average, to complete the entire training paradigm (median = 5.20 h) compared to rhesus macaques (median = 2.98 h) (Table 3), supported by both Kaplan–Meier time-to-event analysis (Log-rank χ^2^ = 31.63 (df = 1), *p <* 0.0001) (Figure 1a) and an independent samples t-test comparing average completion time by group (*p <* 0.0001). Additionally, cynomolgus macaques took significantly longer to complete P-phase training (Figure 1b), Phase-1 training (Figure 1c), and Phase-2 training (Figure 1d) compared to rhesus macaques. Median training times by species for each training phase are presented in Table 3.

**Figure 2:**
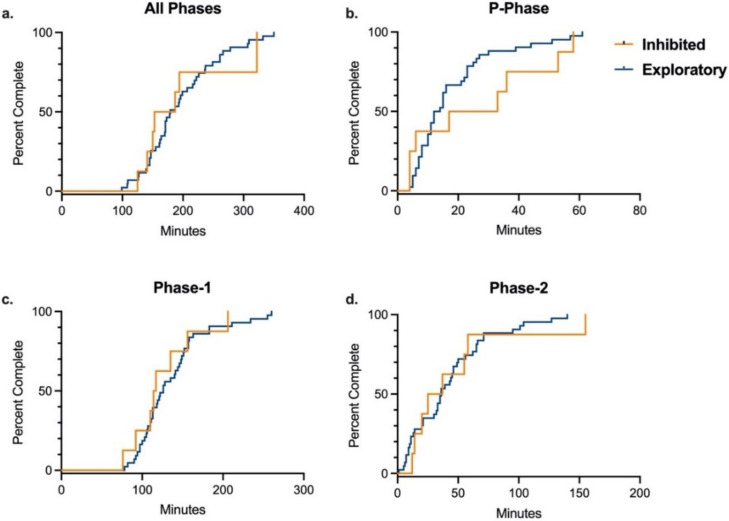
Training Completion Time by Temperament in Rhesus Macaques. Kaplan–Meier time-to-event analysis comparing the time required to successfully complete all phases and individual phases of the training paradigm between inhibited and exploratory rhesus macaques. There were no significant differences between groups for completing (**a**) all phases (Log-rank χ^2^ = 0.03 (df = 1), *p* = 0.86), (**b**) P-Phase (Log-rank χ^2^ = 1.15 (df = 1), *p* = 0.30), (**c**) Phase-1 (Log-rank χ^2^ = 0.50 (df = 1), *p* = 0.50), or (**d**) Phase-2 (Log-rank χ^2^ = 0.26 (df = 1), *p* = 0.61).

**Figure 3:**
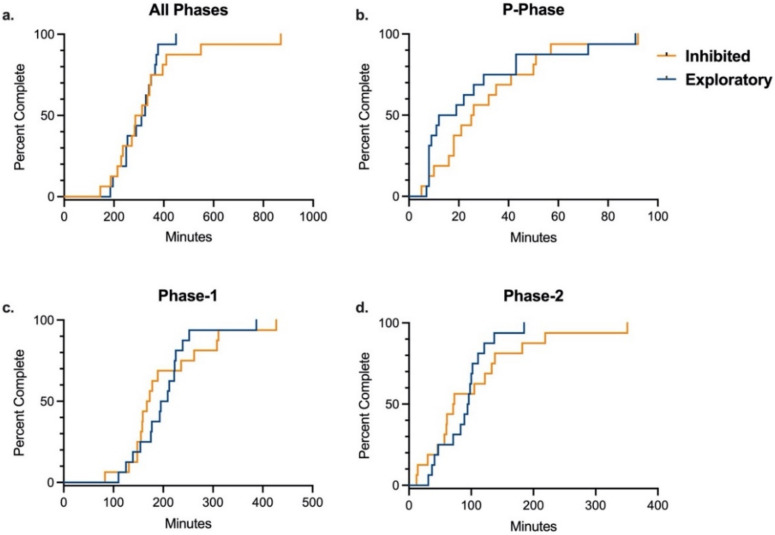
Training Completion Time by Temperament in Cynomolgus Macaques. Kaplan–Meier time-to-event analysis comparing the time required to successfully complete all phases and individual phases of the training paradigm between inhibited and exploratory cynomolgus macaques. There were no significant differences between groups for completing (**a**) all phases (Log-rank χ^2^ = 0.41 (df = 1), *p* = 0.52), (**b**) P-Phase (Log-rank χ^2^ = 0.60 (df = 1), *p* = 0.45), (**c**) Phase-1 (Log-rank χ^2^ = 0.02; (df = 1), *p* = 0.88), or (**d**) Phase-2 (Log-rank χ^2^ = 0.63 (df = 1), *p* = 0.43).

Because significant differences were found in training time between rhesus and cynomolgus macaques, data was stratified by species for analysis of the effects of temperament, sex, and age on training. In each species, there was no difference between inhibited and exploratory animals in the number of total training sessions or total training time required to complete the entire training paradigm, after controlling for sex and age (Table 4 and Table S2). Additionally, there was no difference by temperament in either species in number of training sessions or training time required to complete any of the three individual training phases (Table 4 and Table S2). These results were supported by the Kaplan–Meier analyses comparing time required to complete overall training and individual training phases by temperament in each species (Figure 2 and Figure 3). Age and sex were controlled for in all multivariate logistic regression analyses, and sex was determined to not be an independent predictor of training success for the entire training paradigm or any of its individual phases for either species. Median training time by sex for each species are presented in Table 5. In rhesus macaques, age was determined to not be an independent predictor of training success for the entire training paradigm or any of its individual phases. However, in cynomolgus macaques, logistic regression analysis showed that age was a significant independent predictor in time to complete the P-Phase of training, with older animals completing P-Phase training in less time than younger animals (OR = 0.16, 95% CI = (0.02–0.84), *p =* 0.04). This was supported by independent samples t-test comparing average completion time by age (*p =* 0.03). In contrast, there was no difference in Phase-1, Phase-2, or total training time by age in cynomolgus macaque. Median training time by age for each species are presented in Table 6.

## 4. Discussion

This study explored the influence of various traits on acquisition of successful coping in primates. All animals that were enrolled successfully completed training using a mixed reinforcement paradigm, resulting in animals performing behaviors used for complex medical care in cooperation with caregivers using PRT to maintain the behavioral repertoire. 

### 4.1. Training Differences by Species

Interestingly, we observed significantly different time requirements to complete the same training paradigm between cynomolgus and rhesus macaques suggests there are inherent learning differences and motivational preferences between species [76,77], although the basis for this has yet to be defined. However, this difference of 3 h versus 5 h average total training time in rhesus and cynomolgus macaques, respectively, has little clinical relevance; this is a modest difference in investment in time to train behaviors that will be used in the majority of animals for years. Notwithstanding, understanding the average time commitment required for training a certain animal could have practical implications for study planning related to time management and budgeting. Knowledge of these behavioral differences could prove to be valuable in guiding future model selection, in addition to shaping effective behavioral management practices. From the scientific perspective, deeper understanding of differences in learning behavior influences preclinical model selection to ensure members of the selected species are able to demonstrate the capacity to cooperate, cope, and comply with study procedures in ways that more closely represent the clinical situation. Overall, advancements in our understanding of species-specific differences in training ability can help improve accuracy of study timeline planning, and improve animal welfare by increasing understanding of what is necessary in order to prepare animals to acquire the coping skills necessary to flourish in the research environment. 

### 4.2. Temperament and Behavioral Motivations

We also focused on the interaction between temperament and skills acquisition since behavioral inhibition is associated with an increased risk for stress, and subsequently, anxiety and depression [2,8,9,41,42]. Behaviorally inhibited primates were significantly less likely to accept treats from novel handlers, consistent with vigilance, neophobia, and avoidant tendencies during unfamiliar situations. These results agree with previous research showing that inhibited primates are less likely to directly take treats, and further support the use of the human intruder test to accurately assess temperament [20]. Training can support cognitive processes capable of moderating negative reactivity or modulate fearful reactions to allow instead for engagement with frequent, rewarding, and successful interactions, an adaptation consistent with positive coping that decreases stress. In this study, behaviorally inhibited NHPs successfully performed target behaviors at the same rate as those that were more exploratory, suggesting the early-phase mixed reinforcement used in our program is useful to support skill acquisition and positive coping mechanisms in animals that display high levels of reactivity and distress to novel or unfamiliar objects or people.

Temperament-associated differences in willingness to accept highly palatable treats or other appetitive rewards has important implications related to motivational preferences, which can help inform the design of the most suitable training paradigm for a given animal (e.g., PRT only vs. mixed reinforcement). Previous studies have shown that inhibited primates are significantly less successful in training with a PRT-only based approach, which might be explained by lower motivation to accept positive reinforcers, such as high-valued treats, by behaviorally inhibited animals relative to their more exploratory counterparts [20]. For these inhibited animals that exhibit more fearful, avoidant, hesitant, and relatively neophobic behaviors, the appeal of removing (NR) something unwanted may initially be more motivational than appetitive (PR) rewards [75]. When an unwanted stimulus or condition is subtracted (NR), these animals experience relief, a powerful reinforcer, and trust can be built on the understanding that the trained interaction is safe. When combined sparingly with PRT, NRT can be used to improve training efficacy to reinforce that all interactions with trainers end in a favorable outcome for an animal [68,78,79]. We show that although inhibited animals were less likely to take treats at the initiation of training, they were accepting treats comparably to exploratory animals by the end of the P-Phase of training. Using P-phase as an easy learning phase to modulate reactive tendencies and instead engage animals with frequent rewarding appeared to effectively diminish the effects of temperament in future phases, evident by the absence of significant difference in the time required to complete subsequent training phases. It should be highlighted that during Phase-1 and -2 training, primates were offered a high valued “jackpot” at the end of each training session to offset potential training-related anxiety, foster a positive association with training. Affective state-based cognitive biases can occur when there is no way to buffer or counter-condition a stressful event, and post-training high value rewards can help provide this buffer [80]. It is important to note that even the more inhibited animals were able to benefit from the “jackpot” reward strategy owing to the coping skills and willingness to accept appetitive reinforcers developed during the P-Phase of training and the mixed reinforcement-based training paradigm. These findings suggest that the mixed reinforcement training model, with the incorporation of appropriate familiarization and desensitization steps (in the form of the P-Phase here), supports the learning and development of improved coping strategies required for training success in both exploratory animals, and notably, inhibited animals. The combination of push–pull motivators utilized by mixed-reinforcement may be more effective than either push or pull motivators used in isolation, and should be considered in the design of training programs, especially for use in animals with inhibited temperaments who are less likely to have high success with appetitive rewards alone [79]. 

We did not explore the role of social learning in this analysis. Animals have demonstrated the ability to learn at a faster rate after observing conspecifics performing a task, and animals in this study were housed among animals with varying experience with cooperative tasks [81]. Others have shown the role observational learning has in positively shaping animal perceptions of handlers/trainers [82]. We generally find that animals who observe conspecifics having positive interactions with trainers have reduced overall anxiety behaviors related to close human contact. As such, future studies could investigate the effects of the incorporation of social learning on similarly designed training paradigms. As macaques are highly social animals with hierarchical societies, social rank should also be considered as a variable that may have a potential impact on training efficiency during the learning of complex tasks; rank was not analyzed in this study. Such differences have previously been described in rhesus macaques during simple task training [83,84,85].

### 4.3. Implications for Welfare and Scientific Validity

Despite individual differences in time to complete training, it is important to note that all animals in our study, regardless of temperament, age, sex, or other demographic characteristics, were able to successfully complete the entire training paradigm and acquire the coping skills necessary for appropriately dealing with exposure to potentially adverse medical situations. In biomedical research, ideal sample choice not only includes selecting the best species for a specific question of interest, but also requires the use of a set of subjects that are as representative of the target population as possible. This has most notably led to a recent push for sex-balancing in scientific studies, but also supports the inclusion of other demographic characteristics, such as temperament, to improve generalizability. Temperament-related selection bias can lead to favoring exploratory animals when candidates are chosen based on preconceived perceptions of their trainability, willingness to cooperate, lack of aggression, and other indicators of affective state [86]. Considering temperament is an interaction between individual biological factors and contextual factors, excluding more inhibited animals in favor of those that are more exploratory has the potential to bias results [7,8,9,10,11]. Despite preconceived notions, the present findings show that inhibited animals can be equally successful at learning a complex training task, and should not be excluded from studies based on perceived study suitability. Using behavioral management as a great equalizer, behaviorally inhibited animals can develop equivalent coping skills to protect their welfare and limit stress bias, so that study populations are more generalizable, improving validity and translatability [36].

Welfare is largely affected by the ways that an animal perceives both its environment and its ability to exert control over it, both of which are closely intertwined with temperament, coping skills, and training. This study shows that well-designed cooperative training has the ability to enable animals of any temperament to develop the coping abilities required to adequately deal with routinely experienced medically necessary aversive situations and reduce consequent stress, demonstrating cooperative training’s critical role in fostering overall welfare in captive animals [68]. Further, training often serves as a form of enrichment, giving animals the chance to perform novel physical and cognitive activities, in addition to providing the opportunity to bond with familiar trainers. Captive animals benefit from the opportunity to learn and perform novel behaviors, and training provides this opportunity [21,87].

An important benefit of successful training is the opportunity for animals to express choice and control over their own care, a key component of welfare. Without proper training, animals often develop a learned helplessness to deal with potentially aversive experiences. Learned helplessness, associated with the inability to escape a stressful situation, can induce uncontrollable, unmediated stress and anxiety [88]. Beyond its ethical implications, the welfare benefits and potential reduction in stress introduced by training can help improve the validity of scientific results and the translational value of the research model. Excess stress has been shown to affect a range of research-related physiologic outcome measures such as heart rate, blood pressure, cortisol, and functional immune state in humans [89,90,91,92], and its reduction through training may lead to a more representative model with less stress-related confounding of results. We have demonstrated previously that the ability to make choices to cooperate with a task, learned during training, fosters a sense of control that is protective psychologically and physically in NHPs [68].

### 4.4. Translational Relevance

Beyond its significance for animals in captivity, this research has implications for the management of clinical patients who experience potentially fear-inducing medical procedures for the treatment of a variety of acute and chronic medical conditions. Coping skills training in patients can effectively reduce anxiety and increase compliance with treatment to improve outcomes [93,94,95], but there are gaps in understanding of conditions necessary to support practical treatment options. Primates have become increasingly prominent in understanding biological mechanisms underlying neuropsychiatric disorders, e.g., autism spectrum disorder, emphasizing their relevance in biobehavioral research towards successful interventions [96,97,98]. Behavioral management techniques that foster coping in animals undergoing intensive medical management have strong potential to similarly benefit clinical patients. For example, certain adjustments in management technique for behaviorally inhibited patients, such as reducing the ability to perform avoidance-type behaviors and increasing the opportunity to experience reward while in a controlled setting, could more successfully address the needs of these patients in order to improve coping, foster higher levels of cooperation with medical procedures, and reduce overall stress. 

## 5. Conclusions

Effective coping is essential for animals involved in transplant, metabolic, and infectious disease studies, among others, where they are routinely exposed to medical interventions such as physical examinations, blood sampling, and drug administration. Recognizing and acknowledging individual and species-related differences supports the proper application of training paradigms for successful cooperation and fosters resilience in animal subjects which improves the accuracy of the model to enhance translation and increase animal welfare. 

## Figures and Tables

**Table 1:** Primate Behavioral Ethogram Used for the Classification of Primate Temperament.

Behavior	Description	Association
Active	Moving about, walking, running, climbing, jumping; not lethargic.	E ^1^, I ^2^
Aggressive	High frequency displays; threats.	I *
Alarmed	Fearful; alarm calling; maximizes space to technician.	I *
Bold	Fearless; not restrained or tentative; not timid, shy, or coy.	E *
Calm	Reacts in an even, calm way; is not easily disturbed; not agitated; restful; peaceful.	E *
Confident	Readily explores, investigates novel items.	E *
Depressed	Isolated, withdrawn, sullen, brooding, and has reduced activity.	I *
Curious	Readily explores, eager	E *
Engaged	Interested, interactive, amiable	E
Gentle	Responds to technicians in an easy-going, kind, and considerate manner; not rough or threatening.	E
Playful	Engages in play behavior.	E
Submissive	Displays lower hierarchical behavior; presenting, fear grimace, eye-averting, avoiding, cowering	E, I
Tentative	Timid, shy, hesitant.	I
Vigilant	Alert; ready, attentive, watchful.	I

^1^ E = Exploratory-Type behavior, ^2^ I = Inhibited-Type behavior, * Weighted behavior.

**Table 2:** Training Details for P-Phase.

Sub-Phase	SBP ^1^ Engagement %	Food Reward Offered	Toe-Touching	Trial End Criteria	Sub-Phase Passing Criteria
P-1	0%	Yes	No	Animal takes at least 9 treats by hand OR trial time reaches 4 min	Score of 2. Animal takes food by hand (1 point) post trial AND has an attitude that is engaged, neutral/calm, or curious (1 point) post trial.
P-2	30%	Yes	No	Animal takes at least 9 treats by hand OR trial time reaches 4 min	Score of 2. Animal takes food by hand (1 point) mid-session AND has an attitude that is engaged, neutral/calm, or curious (1 point) mid-session.
P-3	50%	Yes	No	Animal takes at least 9 treats by hand OR trial time reaches 4 min	Score of 2. Animal takes food by hand (1 point) mid-session AND has an attitude that is engaged, neutral/calm, or curious (1 point) mid-session.
P-4	90%	Yes	No	Animal takes at least 9 treats by hand OR trial time reaches 4 min	Score of 2. Animal takes food by hand (1 point) mid-session AND has an attitude that is engaged, neutral/calm, or curious (1 point) mid-session.
P-5	90%	Yes	Yes	Animal is non-reactive to toe touching/holding limbs for 10 s (×3) OR trial time reaches 4 min	Score of at least 2. Animal takes food by hand (1 point) mid-session, has a mid-session attitude that is engaged, neutral/calm, or curious (2 points), or has a mid-session attitude that is submissive or tentative (1 point).

^1^ SBP = Squeeze-back panel.

**Table 3:** Training Time Summary by Species.

Species	All Phases Median (IQR) (Hrs)	P(re) Phase Median (IQR) (Hrs)	Phase 1 Median (IQR) (Hrs)	Phase 2 Median (IQR) (Hrs)
*Rhesus* (*n* = 51)	2.98 (2.45–3.93)	0.23 (0.12–0.42)	2.03 (1.77–2.53)	0.59 (0.23–0.97)
*Cynomolgus* (*n* = 32)	5.20 (3.99–6.02)	0.36 (0.16–0.71)	3.06 (2.57–3.89)	1.53 (0.82–2.03)
*p*-Value (Rhesus v. Cynomolgus)	<0.0001 *	0.0213 *	<0.0001 *	<0.0001 *

* *p* < 0.05.

**Table 4:** Training Time Summary by Temperament.

Species		All Phases Median (IQR) (Hrs)	P(re) Phase Median (IQR) (Hrs)	Phase 1 Median (IQR) (Hrs)	Phase 2 Median (IQR) (Hrs)
*Rhesus* (*n* = 51)	Inhibited (*n* = 8)	2.83 (2.39–4.83)	0.42 (0.08–0.81)	1.93 (1.61–2.51)	0.52 (0.26–0.96)
	Exploratory (*n* = 43)	2.98 (2.45–3.93)	0.20 (0.13–0.38)	2.10 (1.77–2.53)	0.59 (0.23–1.03)
	*p*-Value (I v. E)	0.9021	0.2916	0.5410	0.7460
*Cynomolgus* (*n* = 32)	Inhibited (*n* = 16)	5.00 (3.85–6.40)	0.43 (0.28–0.79)	2.83 (2.50–4.26)	1.20 (0.82–2.28)
	Exploratory (*n* = 16)	5.30 (4.15–6.02)	0.26 (0.13–0.67)	3.37 (2.66–3.74)	1.59 (0.88–1.81)
	*p*-Value (I v. E)	0.4554	0.5713	0.9912	0.5534

**Table 5:** Training Time Summary by Sex.

Species		All Phases Median (IQR) (Hrs)	P(re) Phase Median (IQR) (Hrs)	Phase 1 Median (IQR) (Hrs)	Phase 2 Median (IQR) (Hrs)
*Rhesus* (*n* = 51)	Female (*n* = 24)	3.05 (2.43–3.68)	0.25 (0.17–0.47)	2.04 (1.68–2.52)	0.63 (0.34–1.02)
	Male (*n* = 27)	2.97 (2.55–4.15)	0.18 (0.12–0.42)	2.03 (1.83–2.62)	0.58 (0.22–0.93)
	*p*-Value (Female v. Male)	0.7060	0.4140	0.4277	0.9366
*Cynomolgus* (*n* = 32)	Female (*n* = 11)	4.53 (3.82–6.17)	0.27 (0.13–0.53)	2.78 (2.08–3.70)	1.57 (0.95–2.28)
	Male (*n* = 21)	5.47 (4.15–5.95)	0.42 (0.19–0.72)	3.25 (2.64–4.09)	1.48 (0.73–1.94)
	*p*-Value (Female v. Male)	0.4841	0.2293	0.1170	0.7677

**Table 6:** Training Time Summary by Age.

Species		All Phases Median (IQR) (Hrs)	P(re) Phase Median (IQR) (Hrs)	Phase 1 Median (IQR) (Hrs)	Phase 2 Median (IQR) (Hrs)
*Rhesus* (*n* = 51)	Young (*n* = 26)	2.93 (2.44–4.20)	0.26 (0.13–0.44)	1.99 (1.81–2.47)	0.60 (0.23–1.11)
	Mature (*n* = 25)	3.11 (2.48–3.63)	0.20 (0.12–0.33)	2.14 (1.72–2.67)	0.59 (0.28–0.87)
	*p*-Value (Young v. Mature)	0.8683	0.4141	0.2667	0.2054
*Cynomolgus* (*n* = 32)	Young (*n* = 22)	5.34 (4.15–5.88)	0.47 (0.28–0.84)	3.09 (2.47–4.04)	1.56 (0.95–2.08)
	Mature (*n* = 10)	4.74 (3.76–6.20)	0.18 (0.13–0.33)	3.02 (2.58–3.73)	1.38 (0.70–1.96)
	*p*-Value (Young v. Mature)	0.4004	0.0296 *	0.4794	0.5810

* *p* < 0.05.

## Data Availability

The data presented in this study are available on request from the corresponding author.

## Supplementary Materials

The following supporting information can be downloaded at: https://www.mdpi.com/article/10.3390/biology11030423/s1, Table S1: Primate Demographics; Table S2: Temperament and Training Outcomes.
