# Supplementary material for: Behavioral Management as a Coping Strategy for Managing Stressors in Primates: The Influence of Temperament and Species
Source: Biology (Basel). 2022 Mar 10;11(3):423. doi: 10.3390/biology11030423 (PMC8945664; doi:10.3390/biology11030423)
Supplement: Supplementary file 1 [file biology-11-00423-s001.zip › biology-1630063-supplementary.pdf]

Article

Behavioral Management as a Coping Strategy for Managing Stressors in Primates: The Influence of Temperament and Species

Sierra Palmer [1,2,†], Scott Hunter Oppler [1,2,†] and Melanie L. Graham [1,2,*]

[1] Department of Surgery, University of Minnesota, Minneapolis, MN 55108, USA; palme535@umn.edu (S.P.); opple001@umn.edu (S.H.O.)
[2] Department of Veterinary Population Medicine, University of Minnesota, St. Paul, MN 55108, USA
* Correspondence: graha066@umn.edu
† These authors contributed equally.

Table S1. Primate Demographics.

	Rhesus Macaques	Cynomolgus Macaques	All Animals
**Total Number**	51 (61%)	32 (39%)	83
**Age, years (Median (IQR))**	4.2 (3.0–5.3)	3.5 (3.0–4.5)	4.0 (3.0–4.9)
**Sex (n(%))**			
**Female**	24 (47%)	11 (34%)	35 (42%)
**Male**	27 (53%)	21 (66%)	48 (58%)
**Temperament Classification (n(%))**			
**Inhibited**	8 (16%)	16 (50%)	24 (29%)
**Exploratory**	43 (84%)	16 (50%)	59 (71%)

Table S2. Temperament and Training Outcomes.

	Rhesus Macaque		Cynomolgus Macaque	
	**OR (95% CI)**	***P*-Value**	**OR (95% CI)**	***P*-Value**
**Reward Acceptance**	N/A	N/A	0.08 (0.01, 0.44)	0.012 *
**Total # of Sessions**	4.2 (0.50, 36.0)	0.17	0.75 (0.14, 5.1)	0.73
**Total Training Time**	1.2 (0.15, 6.4)	0.85	0.53 (0.06, 3.9)	0.53
**Total P-Phase Time**	4.9 (0.94, 28.4)	0.06	1.6 (0.29, 8.7)	0.60
**Total Phase-1 Time**	0.75 (0.04, 5.7)	0.80	1.8 (0.4, 9.4)	0.48
**Total Phase-2 Time**	0.46 (0.02, 3.1)	0.50	2.7 (0.54, 16.14)	0.24

OR = Odds Ratio Calculated by Logistic Regression; CI = Confidence Interval; * p <0.05.

Multiple logistic regression model results for the comparison of behavior and training outcomes between inhibited and exploratory rhesus and cynomolgus macaques. Odds ratios correspond to inhibited animals.
